# Intercultural Competence Predicts Intercultural Effectiveness: Test of an Integrative Framework

**DOI:** 10.3390/ijerph19084490

**Published:** 2022-04-08

**Authors:** Stijn Schelfhout, Robin Vandecasteele, Stéphanie De Maesschalck, Fanny D’hondt, Sara Willems, Eva Derous

**Affiliations:** 1 Research Group Vocational and Personnel Psychology, Department of Work, Organisation and Society, Faculty of Psychology and Educational Sciences, Ghent University, H. Dunantlaan 2, 9000 Ghent, Belgium; eva.derous@ugent.be; 2 Department of Experimental Psychology, Faculty of Psychology and Educational Sciences, Ghent University, Henri Dunantlaan 2, 9000 Ghent, Belgium; 3 Interdepartmental Research Group Vocational and Personnel Psychology, Faculty of Psychology and Educational Sciences, Ghent University, Henri Dunantlaan 2, 9000 Ghent, Belgium; 4 Research Group Equity in Health Care, Quality & Safety, Department of Public Health and Primary Care, Faculty of Medicine and Health Sciences, Ghent University, University Hospital Campus Entrance 42, C. Heymanslaan 10, 9000 Ghent, Belgium; robin.vandecasteele@ugent.be (R.V.); stephanie.demaesschalck@ugent.be (S.D.M.); sara.willems@ugent.be (S.W.); 5 Department of Sociology, Faculty of Political and Social Sciences, Ghent University, Sint-Pietersnieuwstraat 41, 9000 Ghent, Belgium; fanny.dhondt@ugent.be; 6 Centre for the Social Study of Migration and Refugees, Ghent University, H. Dunantlaan 2, 9000 Ghent, Belgium

**Keywords:** intercultural traits, intercultural attitudes and worldviews, intercultural capabilities, intercultural competence, intercultural effectiveness, multicultural personality, ethnocentrism, ethnorelativism, cultural intelligence, cultural self-efficacy

## Abstract

Why does someone thrive in intercultural situations; while others seem to struggle? In 2014, Leung and colleagues summarized the literature on intercultural competence and intercultural effectiveness into a theoretical framework. This integrative framework hypothesizes that the interrelations between intercultural traits, intercultural attitudes and worldviews, and intercultural capabilities predict the effectiveness with which individuals respond to intercultural situations. An empirically verified framework can contribute to understanding intercultural competence and effectiveness in health care workers, thus contributing to more equity in health care. The present study sets out to test this integrative framework in a specific health care context. Future health care practitioners (*N* = 842) in Flanders (Belgium) were questioned on all multidimensional components of the framework. Structural equation modeling showed that our data were adequate to even a good fit with the theoretical framework, while providing at least partial evidence for all hypothesized relations. Results further showed that intercultural capabilities remain the major gateway toward more effective intercultural behavior. Especially the motivation and cognition dimensions of cultural intelligence seem to be key factors, making these dimensions an excellent target for training, practical interventions, and identifying best practices, ultimately supporting greater intercultural effectiveness and more equity in health care.

## 1. Introduction

In our ever globalizing world of today, interculturalism is becoming the norm. People select and communicate cultural information (for instance regarding ethnicity, race, religion and nationality) according to the situation and their personal interests [1]. Moreover, in health care, such communication can define interactions between practitioners and patients with diverse cultural background [2,3,4,5]. This intercultural and interacting health care setting can instigate specific challenges, especially as these interactions can threaten equity in health care [6,7,8]. Literature features quite some variance in how equity in health care is described [9]. Lane and colleagues reviewed this equity literature and suggested a practically operationalized definition of equity in health care depending on the specific application [10]. Following Lane and colleagues [10], the present study thus defines equity in health care as follows: Reducing disparities toward access to and use of health care services for groups of people with different nationality or ethnic backgrounds. As a prime example of such a challenge to equity in health care, ethnic prejudice is described as the unjustified or incorrect attitude toward individuals based solely on their membership of certain groups like race or nationality [11,12]. As a consequence, ethnic prejudice can cause inequity in health care as people from different races or nationalities are receiving suboptimal treatment, resulting in less favorable therapy outcomes and poorer health altogether [13,14,15].

Recently, literature aimed at studying problems such as ethnic prejudice in a broader framework of intercultural interaction [16,17]. Indeed, why do some people thrive in intercultural situations, while others seem to struggle? To answer this question, Leung and colleagues proposed an integrative framework that theorizes how the intercultural competence of individuals leads to effective behavior in intercultural situations [18]. The model is developed based on an extensive review of theoretical and empirical developments in the intercultural competence literature. As such, the model describes how personality, world views (including attitudes), and knowledge of different cultures interact toward effectiveness in intercultural situations. The framework benefits from a broad scope that can be applied to a multitude of settings including health care. The authors have explicitly stated that researchers should explore how the components of intercultural competence interrelate regarding their effect on intercultural effectiveness, instead of viewing the different components as independent predictors.

For sure, the merit of this framework cannot be underestimated in summarizing the extant literature on intercultural competence and its (theorized) effects on intercultural effectiveness. However, despite the authors’ call for empirical validation, to this date and to our knowledge, the theoretical framework has not been empirically verified in full. Toward a specific setting like health care, such a verification can provide us with an invaluable basis to further study and even remedy urgent problems like ethnic prejudice that threaten equity in health care.

The present study in Flanders (i.e., the largest region in Belgium) therefore aims to provide empirical verification of the framework on intercultural competence by investigating how the components of intercultural effectiveness interrelate in exerting their effects on intercultural effectiveness. In doing so, the present study also focuses on identifying which specific framework components are responsible for intercultural effective behavior in a health care setting. Following the advice of Brottman and colleagues, these components can become the future target of training, intervention, and evaluation, in order to establish a compendium of best health care practice and education [19]. For instance, the present study is conducted within the EdisTools project. The project wants to improve intercultural effectiveness of practitioners toward clients in key domains of society like health care by developing a set of digital tools for training and education.

### 1.1. The Framework of Intercultural Competence and Intercultural Effectiveness

Dias and colleagues describe intercultural competence as a multifaceted and complex concept, that combines the knowledge and skills needed to perform effectively and appropriately when interacting with others who are culturally different [20]. Literature already features a plethora of articles on the nature of this knowledge and these skills as intercultural competence is becoming key in important areas of human interaction such as education and healthcare. Leung and colleagues have summarized this literature into an integrative framework that theorizes how three components of intercultural competence interact to achieve intercultural effectiveness [18]. First, intercultural traits indicate how well an individual’s personality is able to handle intercultural situations [21,22]. Second, intercultural worldviews indicate how individuals perceive (information from) other cultures [23,24,25]. Finally, intercultural capabilities represent an amalgam of what a person can undertake in order to be effective in an intercultural situation such as a job interview where participants have different cultural backgrounds [26,27].

#### 1.1.1. Intercultural Traits

The Big Five (extraversion, agreeableness, openness, conscientiousness, and emotional stability) of human personality are known to be good predictors of how successful individuals are in actually evaluating (intercultural) situations and also appropriately responding to these situations [28]. For instance, Shaffer and colleagues reported that expats showed a positive relation of *r* = 0.24 between emotional stability and work adjustment and a negative relation of *r* = −0.27 between emotional stability and cognitions about early withdrawal from the job [29]. Toward multicultural settings, van der Zee and van Oudenhoven have proposed a contextualized model of five multicultural personality traits that have incremental validity above and beyond the original Big Five [22,30,31]. Cultural empathy thus refers to empathy toward the feelings, thoughts, and behavior of individuals with a diverse culture. Emotional stability refers to the ability to stay calm despite novel and stressful intercultural conditions. Flexibility reflects an aptitude in interpreting novel intercultural situations as a positive challenge, while also adapting to these situations. Open mindedness represents an unprejudiced disposition toward differences in culture. Finally, social initiative refers to actively approaching intercultural social situations while also taking the initiative in these situations. A high score on these multicultural personality dimensions was found to be predictive regarding the successful adjustment of employees, students, migrants, and expats in their intercultural work or study activities, even when controlling for the original Big Five [32,33,34]. Recently, a Short Form of the Multicultural Personality Questionnaire (i.e., SF-MPQ with 40 items) [35] has also been validated for use in health care-related contexts [36].

The predictive value of traits as constructs is partly explained by their stability, as personality traits are known to be quite stable over the lifespan, especially past childhood [37,38]. For instance, literature indicates that personality traits are associated with ethnocentric world views like ethnic prejudice and right-wing authoritarianism [39,40]. Personality traits are also associated with intercultural capabilities like cultural intelligence [41,42]. The framework of Leung and colleagues [18] thus hypothesizes the following relations regarding intercultural traits, intercultural worldviews, and intercultural capabilities (see further).

**H1.**  *Higher scores on intercultural traits predict a more ethnorelative world view (As a standalone investigation, the present study’s cross-sectional data do not allow for a strict causal interpretation of the results. However, (a) as the hypotheses are investigated through regression SEM analyses and (b) as the hypotheses tested are the result of prior extensive theoretical and empirical research in extant literature, the present study’s hypotheses are posited as predictions.).*

**H2.**  *Higher scores on intercultural traits predict higher scores on intercultural capabilities*.

#### 1.1.2. Intercultural Worldviews

Intercultural worldviews address how individuals perceive information from other cultures [23,24,25]. As an example, the Developmental Model of Intercultural Sensitivity places ethnocentrism and ethnorelativism at both ends of a continuum that represents increasing intercultural competence [43,44]. An individual with an ethnocentric disposition almost exclusively observes the world through the looking glass of its own culture [45]. An individual with an ethnorelative disposition emphasizes the complexities and contradictions of many different countries and cultures instead [46]. Research by Hammer has shown that a score on this continuum can predict reduced intercultural anxiety, satisfaction with studying abroad, the number of intercultural friends, and inclusion policies on staff recruiting [47,48]. In health care specifically, Kaya and colleagues showed that Turkish nursing students showed a negative correlation between intercultural sensitivity and ethnocentrism of about *r* = −0.40 [49]. Based on the already extensive literature on the topic, Leung and colleagues hypothesized that an ethnorelative world view has a positive effect on intercultural capabilities [18,50,51].

**H3.**  *A more ethnorelative world view predicts higher scores on intercultural capabilities.*

#### 1.1.3. Intercultural Capabilities

Intercultural capabilities focus on what a person can do to be effective in an intercultural interaction like showing knowledge of other cultures [52]. One often-used conceptualization of intercultural capabilities is (inter)cultural intelligence [53]. Cultural intelligence can be described as a set of adaptable properties that allows an individual to effectively manage intercultural situations [54]. Literature already reached consensus that cultural intelligence at least features a (meta) cognitive dimension (i.e., knowledge about different cultures), a motivational dimension (i.e., motivation to interact with people from different cultures), and a behavioral dimension (i.e., knowledge about how to act in intercultural interactions) [52,55,56,57,58]. Research on these dimensions with adolescents showed a positive relation with contact and cooperation and multiculturalism in both immigrant and non-immigrant students [59]. In health care specifically, Harrison and colleagues found that intercultural competence of health care professionals and effective practitioner-patient engagement are strongly related regarding ethnic minority populations [60].

These observed effects are possible due to the adaptable nature of intercultural capabilities. In other words, capabilities like cultural knowledge are teachable and thus trainable, making these capabilities a prime target for training and education interventions in order to improve an individual’s intercultural competence and eventually also the individual’s effectiveness in intercultural situations [61]. In the framework of Leung and colleagues [18], intercultural capabilities are thus hypothesized to have a positive effect on intercultural effectiveness.

**H4.**  *Higher scores on intercultural capabilities predict higher scores on intercultural effectiveness.*

### 1.2. Intercultural Effectiveness

The goal of intercultural competence in an individual is for that individual to exhibit effective behavior in intercultural situations. The downside of studying actual behavior lies in the fact that you can observe the behavior, but you cannot directly tell why individuals exhibit said behavior. As an alternative, literature harbors classic models that explain human behavior through different psychological (multidimensional) constructs that can be measured instead of actual behavior. For instance, the Social Cognitive Theory posits that human behavior can be explained through the determinants of self-efficacy belief (i.e., can I make it happen?), outcome expectation (i.e., what will be the outcome if it happens?), and goal representation (i.e., what will I gain if it happens?) [62]. Self-efficacy belief is regarded the most essential component as an individual with low self-belief will have a very low chance to initiate the behavior to begin with [63]. As such, previous studies have already indicated a strong connection between general self-efficacy and behavior [64,65]. For instance, meta-analytic evidence on the theory of planned behavior shows that as perceived behavioral control, self-efficacy can have major average correlations with actual behavior of about *r* = 0.46 [66]. More specifically, literature has also already established a relationship between cultural intelligence and self-efficacy [67,68]. For example, Quine and colleagues reported that Canadian nursing students self-rated their cultural self-efficacy as moderate when dealing with diabetes patients from aboriginal ancestry [69]. Moreover, Quine and colleagues also showed that higher cultural self-efficacy was related to greater intercultural communication and lower intercultural anxiety, with an explained variance of about 33 to 35% [69].

Cultural self-efficacy can be operationalized as a multidimensional construct. As an example, Briones and colleagues suggest a five-dimensional construct as operationalized in the cultural self-efficacy scale for adolescents or CSES-A [70]. The process dimension indicates how well individuals estimate their own ability to process information about other cultures. The mix dimension indicates how well individuals estimate they would mix within other cultures. The cope dimension indicates how well individuals estimate their ability to cope with homesickness and separation from loved ones. The understanding dimension indicates how well individuals estimate their ability to understand other ways of life. Finally, the language dimension indicates how well individuals estimate they are able to learn and understand other languages. Both the multidimensional construct as well as the instrument are already quite common in health care literature [71,72,73].

### 1.3. Present Study

The present study aims to empirically verify the integrative framework of Leung and colleagues in a health care setting [18]. To this extent, H1 to H4 are tested in a structural equation model (SEM), using a multidimensional approach. For the present study, a multidimensional approach is important as such an approach can pinpoint how exactly the intercultural competence triangle (through multidimensional cultural intelligence) relates to intercultural effectiveness (through multidimensional cultural self-efficacy). Such interrelations are key to establishing future training, intervention, and evaluation targets, in order to establish a compendium of best health care practice and education [19].

The framework by Leung and colleagues does not elaborate on two relations: (a) does multicultural personality have a direct effect on intercultural effectiveness and (b) does an individual’s world view have a direct effect on intercultural effectiveness [18]?

First, literature already reports some evidence of direct effects of various dimensions of multicultural personality on intercultural effectiveness. Indeed, Ward and Fischer found that flexibility, social initiative, and emotional stability had a direct effect on the general adjustment of international students in New-Zealand [42]. Moreover, the general personality trait openness to experience was found to have a direct effect on adaptive performance [74] and job performance [75], albeit partially mediated through the motivation dimension of cultural intelligence. As a specific example from health care, Herrera and Owens found that higher post-traumatic stress disorder (PTSD) in US service members was predicted by higher levels of open mindedness and lower levels of flexibility and emotional stability [21]. As such, we have examined the possibility of extending the proposed framework of Leung and colleagues with a direct relation between (multicultural) personality traits and intercultural effectivity [18].

**H5.**  *Higher scores on intercultural traits directly predict higher scores on intercultural effectiveness.*

Second, literature also reports direct effects of world views on intercultural effectiveness. For sure, an ethnocentric world view in health care professionals is known to lead to negative outcomes like misdiagnosis, mistreatment, and undertreatment in culturally diverse individuals [76]. For instance, an experimental design in a health context revealed that the willingness to interact with an intercultural health professional correlated negatively with ethnocentrism, *r* = −0.19 [77].

**H6.**  *A more ethnorelative world view directly predicts higher scores on intercultural effectiveness*.

**Figure 1:**
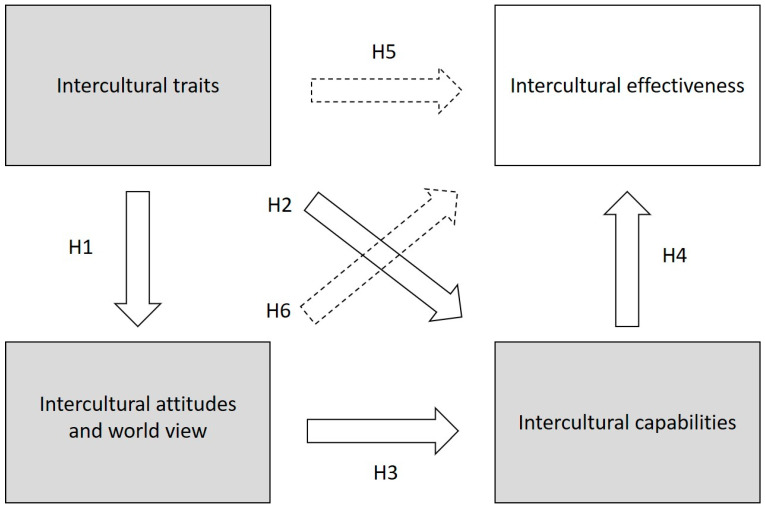
Hypotheses summary: test of an integrative framework. Note: The grey squares represent intercultural competence. The operationalizations of intercultural competence and effectiveness are presented in italic. H1, H2, H3, and H4 represent the empirical test of the integrative framework as proposed by Leung et al., 2014. H5 and H6 represent additional hypotheses in dashed arrows. All hypothesized relationships are expected to have positive effects.

Figure 1 summarizes the presented hypotheses toward the empirical verification of the integrative framework of intercultural competence and effectiveness.

## 2. Materials and Method

### 2.1. Data and Procedure

The data were gathered in the context of the EdisTools project, a large Strategic Basic Research project in Belgium (Flanders), subsidized by Scientific Organizations Flanders. This EdisTools project aims to map out interculturalism and prejudice in four different professional settings, which are also embedded in legislation as constitutional rights: education, health care, housing, and work. The project focuses on the intercultural effectiveness of the ethnic majority (i.e., Belgian) service provider (i.e., teachers, doctors, estate agents and employers) toward the ethnic minority client (i.e., learners, patients, potential tenants, and job applicants respectively). This project was approved by the Ethical Medical Committee of Ghent University Hospital, with approval number BC-07577. Practically, the project wants to improve intercultural effectiveness in key domains of society like health care by developing a set of digital tools for training and education. As the present study also focuses on identifying key pathways of intercultural competence for purposes of future education and training in a medical context, a student sample of undergraduate medical students was deemed appropriate. In April 2020 and April 2021, all students in the 1st year of Medical School, in the pre-Master program in Management and Organization of Healthcare, and in pre-Master Program in Health Promotion at the Faculty of Medicine and Health Sciences of a large Belgian University with a top 100 rating in the Academic Ranking of World Universities (ARWU; see also [78]) were therefore invited to participate in the present study and fill out an online survey. All students gave informed consent prior to filling out the survey. Response rate was 76% (*N* = 842), with 74% female students with an average age of *M* = 22.03 (*SD* = 3.28), *Mdn* = 21 (i.e., 69% of the student population aged between 20 and 22). The vast majority of the students (96%) had Belgian nationality. These numbers are in line with population statistics as 78% of the Flemish health care practitioners are women [79], while 92% of the total Belgian population has the Belgian nationality [80]. Slightly less than 11% of the students reported a parent or grandparent with a non-Belgian nationality. Furthermore, 41% of the students reported (close) friends, close colleagues, or family with a migration background.

### 2.2. Measures

#### 2.2.1. Intercultural Traits

Intercultural traits were conceptualized as multicultural personality dimensions and operationalized using the SF-MPQ [35]. The SF-MPQ has five subscales with eight items each and a recurring instruction: “to what extent do the following statements apply to you?” Participants have to respond to this question on a 5-point Likert scale from 1 (totally not applicable) to 5 (completely applicable). For cultural empathy (MPQ-CE, *M* = 4.18, *SD* = 0.43, *α* = 0.80), the subscale included items like “Is a good listener”. For flexibility (MPQ-FX, *M* = 2.45, *SD* = 0.71, *α* = 0.87), the subscale included items like “Works according to plan” (reverse coded). For social initiative (MPQ-SI, *M* = 3.32, *SD* = 0.64, *α* = 0.85), the subscale included items like “Takes initiative”. For emotional stability (MPQ-ES, *M* = 3.08, *SD* = 0.71, *α* = 0.85), the subscale included items like “Is insecure” (reverse coded). Finally, for open mindedness (MPQ-OP, *M* = 3.45, *SD* = 0.53, *α* = 0.78), the subscale included items like “Has broad range of interests”. Important to note, although the five dimensions of the MPQ are correlated [33], the MPQ does not support an overarching general dimension.

#### 2.2.2. Intercultural Attitudes and Worldviews

Intercultural attitudes and worldviews were conceptualized through the ethnocentric-ethnorelative continuum (EC-ER) and operationalized using six items adapted from the European Social Survey [81] on a ten-point Likert scale (*M* = 6.67, *SD* = 1.37, *α* = 0.81). As an example, participants had to respond to items like “Would you say it is generally bad or good for the economy that people come to live here from other countries?” A higher or lower score on the EC-ER scale respectively represents a more ethnorelative or ethnocentric world view.

#### 2.2.3. Intercultural Capabilities

Intercultural capabilities were conceptualized as cultural intelligence (CQ) and operationalized using the Adapted Self-Report CQ Scale [56]. The Adapted Self-Report CQ Scale has four subscales with six items each. For each item, participants had to indicate to which degree they agreed to the presented statement on a 5-point Likert scale (1—strongly disagree to 5—strongly agree). For motivation (CQ-MOT, *M* = 3.88, *SD* = 0.53, *α* = 0.79), the subscale included items like “It’s fun for me to interact with people from other cultures”. For cognition (CQ-COG, *M* = 2.86, *SD* = 0.67, *α* = 0.87), the subscale included items like “I can describe how parents treat their children in various cultures”. For metacognition (CQ-META, *M* = 3.53, *SD* = 0.56, *α* = 0.75), the subscale included items like “When I meet people from another culture, I try to find out how to act appropriately in that culture”. And finally, for behavior (CQ-BEH, *M* = 3.57, *SD* = 0.48, *α* = 0.68), the subscale included items like “If there is a misunderstanding between people from different cultures, I try to clear it up”.

#### 2.2.4. Intercultural Effectiveness

Intercultural effectiveness was conceptualized as cultural self-efficacy (CSE) and operationalized using the CSES-A [70]. The Brottman and colleagues review on cultural competence in health care mentioned that CSES-A was the most frequent (i.e., about 6%) assessment tool found in the selected papers [19,70]. This scale has five subscales with a varying amount of items. For each item, participants had to answer to a recurring question on a five-point Likert scale (1—cannot do at all to 5—certainly can do): “In the situations posed below, mark to what extent you feel capable of carrying them out using the options given”. For process (CSE-process, five items, *M* = 3.61, *SD* = 0.53, *α* = 0.74), the subscale included items like “Speaking to people from a different culture, I can realize what I know about that culture”. For mix (CSE-mix, eight items, *M* = 3.82, *SD* = 0.56, *α* = 0.87), the subscale included items like “If I lived in a different culture, I would be able to make new friends”. For cope (CSE-cope, four items, *M* = 2.90, *SD* = 0.86, *α* = 0.88), the subscale included items like “If I lived in a different culture, I would be able to overcome loneliness”. For understanding (CSE-US, five items, *M* = 3.66, *SD* = 0.62, *α* = 0.78), the subscale included items like “Approaching a different culture, I can understand other religious beliefs”. And finally, for language (CSE-LAN, three items, *M* = 3.17, *SD* = 0.85, *α* = 0.86), the subscale included items like “Approaching a different culture, I can learn a language different from mine”.

### 2.3. Analyses

Analyses were conducted using a SEM set up by means of the lavaan package in R [82,83]. For an overview on SEM-analyses, we also refer to Rosseel [82,84]. For an overview regarding modifying and reporting SEM, we refer to Ullman and Bentler [85] and Schreiber and colleagues [86]. For analyzing model fit, we made use of common guidelines in literature by Hu and Bentler [87], Kenny and colleagues [88], and Rosseel [82]. As SEM literature shows quite some controversy regarding cherry picking fit indices, the present study features a small, but commonly reported battery of fit indices. The chi-square test is the primary test for SEM. A model has a good fit if the chi-square test does not reach significance at the *α* = 0.05 level. However, literature has reached consensus that this test is too strict. As such, we report a set of three other fit indices, that are generally considered as complementary [89]. The first index used is the Root Mean Square Error of Approximation (RMSEA). The RMSEA renders an absolute fit value (i.e., no comparison to other models), of which the 90% confidence interval (CI) should have a lower value no higher than 0.05 and a higher value lower than 0.08. The second index used is the Standardized Root Mean Square Residual (SRMR). The SRMR also renders an absolute fit value, which should be lower than 0.08. Finally, the third index used is the Comparative Fit Index. The CFI is an incremental (relative) index that compares models to the (worst possible) null model. In a model with an adequate fit, the CFI should be higher than 0.95. However, if the RMSEA of the baseline model is too good (i.e., RMSEA < 0.158), the CFI estimate will be too low, rendering the CFA useless as a measure of fit [88]. Literature does recommend caution in interpreting these rules of thumb too rigorously. Researchers should primarily aim for fit indices that show similar results, while also scanning for other indications of bad fit like non-significant parameters at different levels of significance (The *p*-values are reported with three decimals to allow for model respecification at different significance levels.). For a concise overview on additional SEM fit literature, we also refer to Kenny [90].

A SEM analyses consists of two major parts. The structural part presents path analyses on how variables interact with each other. The latent part allows to assess how individual items load on latent constructs, as is the case in confirmatory factor analyses (CFA). For the present study, we are primarily interested in the structural part, as specific regression analyses will allow us to test H1 through H6 and the instruments are already validated in various studies (see Section 1 Introduction). However, as the validity of data is always dependent on both population and instrument, we have used CFA to evaluate the construct validity of the data, generated by the full battery of instruments. As the full models are quite extensive, we have provided two variance–covariance matrices as a data supplement, allowing full replication of our results.

## 3. Results

### 3.1. Preliminary Analysis: First Order Correlations and CFA

Table 1 shows the first order Pearson correlation coefficient for all variables. Important to note, both the total scales as well as the subscales (if present) are included. To assess the construct validity of our data, we performed a CFA on the following (sub-)scales and their theorized individual internal structure: MPQ (CE, FX, SI, ES, and OM), EC-ER, four subscales of CQ (MOT, COG, META and BEH) and five subscales of CSE (process, mix, cope, US, and LAN). The model converged after 128 iterations, with χ^2^ (*N* = 842, 4333) = 12301.53, *p* < 0.001. The set of fit indices showed a somewhat mixed result. Both the RMSEA = 0.047, 90% CI [0.046, 0.048], *p* = 1.000 as well as the SRMR = 0.068 indicated an adequate to even a good fit, while the CFI = 0.77 indicated a poor fit. However, closer inspection of the base null model (which the CFA model is compared against to calculate the CFI) revealed that the null model rendered a RMSEA = 0.097, which violates the condition that the null model has to have an RMSEA > 0.158. As such, the CFI is no longer considered informative. As the CFA model fits the data adequately, we therefore conclude that our obtained data allow us to draw valid conclusions regarding the present study’s hypotheses.

### 3.2. Model 1: Saturated Model

We started our analyses with a model featuring the total scale scores, without subdimensions. Model 1 thus features the scores on the five MPQ scales (i.e., CE, FX, SI, ES and OM), as well as the total scores on EC-ER, CQ, and CSE. As such, Model 1 aims to test all hypotheses (H1 to H6) at a structural path level, without subscales. Model 1 converged after 40 iterations, using maximum likelihood as means of estimation. Model 1 is a fully saturated model, meaning that all variances, covariances, and means of the observed variables are perfectly reproduced. The chi-square test on the saturated model renders χ^2^ (*N* = 842, 0) = 0. Our set of selected fit indices also renders perfect fitting values, which are inherent to saturated models: RMSEA = 0, SRMR = 0 and CFI = 1. Table 2 shows all regressions within Model 1. Model 1 also rendered an explained variance of R^2^ = 0.17 for EC-ER, R^2^ = 0.30 for CQ and R^2^ = 0.39 for CSE. Although the model is saturated and thus inherently has the best possible fit indices, the model still harbors non-significant pathways. These pathways have to be removed in Model 2 in order to evaluate the present study’s hypotheses.

### 3.3. Model 2: Structural Model

**Figure 2:**
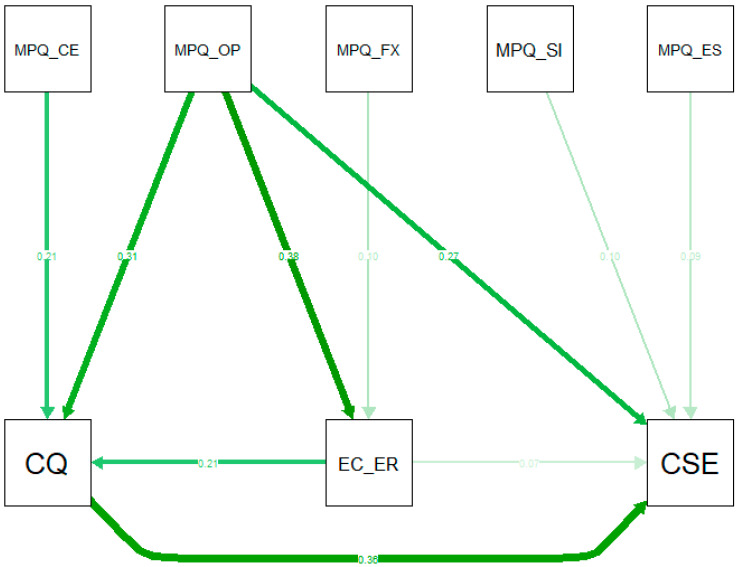
Graphical representation of Model 2. Note: MPQ = multicultural personality questionnaire, CE = cultural empathy, FX = flexibility, SI = social initiative, ES = emotional stability, OP = open mindedness, EC_ER = ethnocentric–ethnorelative continuum, CQ = cultural intelligence, CSE = cultural self-efficacy, US = understanding, LAN = language. All reported relationships have positive and significant effects of at least *p* < 0.05.

Figure 2 shows the structural Model 2. Model 2 converged after 32 iterations. The chi-square test for Model 2 is non-significant, χ^2^ (*N* = 842, 8) = 13.85, *p* = 0.086, which indicates that Model 2 has an adequate fit, even when testing against the most conservative test possible. For reasons of comparability, we also report the RMSEA = 0.029, 90% CI [0.000, 0.055], *p* = 0.901, the SRMR = 0.020, and the CFI = 0.993. Table 3 shows all regressions within Model 2. Model 2 also rendered an explained variance of R^2^ = 0.17 for EC-ER, R^2^ = 0.30 for CQ and R^2^ = 0.39 for CSE. However, Model 2 did not include the subscales for CQ and CSE. These subscales are crucial to assess how intercultural competence has an effect on intercultural effectiveness.

### 3.4. Model 3: Structural Model with Subscales

For Model 3a, we started from the full set of subscale scores: EC-ER, MPQ (i.e., CE, FX, SI, ES, and OM), CQ (i.e., CQ-MOT, CQ-COG, CQ-META, and CQ-BEH), and CSE (i.e., CSE-process, CSE-mix, CSE-cope, CSE-US, and CSE-LAN). However, the model did not show adequate fit measures. First, the chi-square test was significant χ^2^ (*N* = 842, 6) = 306.036, *p* < 0.001. Second, the set of fit indices indicated an insufficient fit of the model with the RMSEA = 0.244, 90% CI [0.221, 0.267], *p* < 0.001, the SRMR = 0.049, and the CFI = 0.892.

As such, Model 3b removed all non-significant pathways at the *α* = 0.05 significance level and again at the *α* = 0.01 significance level to improve the model fit in subsequent iterations. The significance of the pathways is evaluated using the z-score, which is a function of both the unstandardized effect as well as the standard error of the effect. This respecified Model 3b showed a significant chi-square test, χ^2^ (*N* = 842, 47) = 141.857, *p* < 0.001. However, our set of fit indices showed an adequate to good fit of Model 3b, with the RMSEA = 0.049, 90% CI [0.040, 0.058], *p* = 0.557, the SRMR = 0.042, and the CFI = 0.966. Table 4 shows all regressions within Model 3b. Table 5 shows the explained variances for all endogenous (sub) scales.

To finalize our analyses, we inspected the modification indices of Model 3b. Modification indices present information on possible model improvements by presenting alternative pathway relations that can possibly improve the model. Conservatively, we only considered modification indices above 10.83, as this threshold corresponds with an effect size of which the *p*-value is lower than 0.001. Analyses rendered eight possible extensions to the model, with a maximum modification index of 20.51. We did not observe any pathways that were linked to our hypotheses, which strengthens the validity of Model 3b. We did observe four possible additions in which CQ subscales are regressed on other CQ subscales, and four possible additions in which CQ subscales were regressed on CSE subscales. Especially the latter suggestions are interesting, suggesting that self-efficacy can also have an (recursive) effect on intercultural competencies. However, for the present study, we do not have sufficient theoretical grounds to add these possible additions to the final model.

As we now have adequate fit measures on a model with specific subscales and no non-significant regressions in Model 3b, we can assess our hypotheses based on the regressions of the model.

### 3.5. Hypotheses Evaluation

For H1 (i.e., higher scores on intercultural traits predict an ethnorelative world view) Table 4 shows that the regression of EC-ER on both MPQ-OP as well as MPQ-FX is significant. We therefore conclude, we have at least partial empirical evidence that supports H1.

For H2 (i.e., higher scores on intercultural traits predict higher scores on intercultural capabilities), Table 4 shows that (a) the regression of CQ-MOT on MPQ-CE and MPQ-OP is significant; (b) the regression of CQ-COG on MPQ-OP is significant; (c) the regression of CQ-META on MPQ-CE and MPQ-OP is significant; and (d) the regression of CQ-BEH on MPQ-CE is significant. In sum, two out of five MPQ dimensions are positively related to all four dimensions of CQ. We therefore conclude that we have at least partial empirical evidence that supports H2.

For H3 (i.e., a more ethnorelative world view predicts higher scores on intercultural capabilities), Table 4 shows that the respective regressions of CQ-MOT, CQ-MET, and CQ-BEH on EC-ER are significant. In sum, EC-ER was positively related to three out of four dimensions of CQ. We therefore conclude that we have at least partial empirical evidence that supports H3.

For H4 (i.e., higher scores on intercultural capabilities predict higher scores on intercultural effectiveness), Table 4 shows that (a) the regression of CSE-process on CQ-MOT and CQ-COG is significant; (b) the regression of CSE-mix on CQ-MOT is significant; (c) the regression of CSE-cope on CQ-MOT and CQ-COG is significant; (d) the regression of CSE-US on CQ-MOT and CQ-COG is significant; and (e) the regression of CSE-LAN on CQ-COG is significant. In sum, two out of four dimensions of CQ are positively related to all five dimensions of CSE. We therefore conclude that we have at least partial empirical evidence that supports H4.

For H5 (i.e., higher scores on intercultural traits predict higher scores on intercultural effectiveness), Table 4 shows that (a) the regression of SE-process on MPQ-CE and MPQ-OP is significant; (b) the regression of SE-mix on MPQ-OP and MPQ-SI is significant; (c) the regression of CSE-cope on MPQ-ES is significant; (d) the regression of CSE-US on MPQ-CE is significant; and (e) the regression of CSE-LAN on MPQ-OP is significant. In sum, four out of five dimensions of the MPQ are positively related to all five dimensions of CSE. We therefore conclude that we have at least partial empirical evidence that supports H5.

Finally, for H6 (i.e., a more ethnorelative world view predicts higher scores on intercultural effectiveness), Table 4 shows that the regression of CSE-US on EC-ER is significant. As EC-ER is only related to one out of five sub-dimensions of CSE, we conclude that we have some partial empirical evidence that supports H6. 

For replication of our analyses, two variance-covariance matrices were added as Supplementary Materials.

## 4. Discussion

Today, effective intercultural interaction is an essential part of ensuring equity in health care [2,3,4,5,6,9,10,16,17]. Negative outcomes like prejudice are responsible for suboptimal treatment of people from different race or nationality, resulting in less favorable therapy outcomes [13,14,15]. Leung and colleagues have summarized the literature on the effectiveness of intercultural interaction (i.e., inside and outside of health care) into a theoretical framework [18]. This integrative framework hypothesizes that intercultural competence determines how effective individuals handle intercultural situations. This competence is determined through the interrelations between three key framework components: intercultural traits, intercultural interactions and worldviews, and intercultural capabilities. Although the theoretical importance of this framework cannot be underestimated, the authors themselves have called for empirical verification. Addressing this call, the present study empirically tested the integrative framework on intercultural effectiveness, rather than just studying parts of it. As such, we conceptualized all framework components using validated multidimensional constructs, operationalized with appropriate instruments. Ultimately, a validated framework can identify which framework components are responsible for intercultural effective behavior. Moreover, these components can become the target of training, intervention, and identification of best practices to ensure all ethnic groups of patients with different nationalities are treated fairly toward more equity in health. As an example, the EdisTools project will use these findings to develop a set of digital tools for training and education in health care.

A fairly large sample of (future) health care practitioners from the faculty of Medicine and Health Sciences of a large Western-European university filled out the online survey including the operationalized constructs. Using structural equation modeling on the data obtained, we tested the general fit and the hypothesized relations from the framework. In doing so, we also tried to explore how exactly the different components of the framework are interrelated and if we could possibly extend the model.

For the first time in literature, to the best of our knowledge, the present study empirically verified the full theorized framework proposed by Leung and colleagues (2014), as the model fitted our data quite well. First, the present study replicated the (latent) structure of four independently developed instruments, thus validating our data toward the current research. Second, the battery of fit indices indicated an adequate fit, for models with and without subscales (see also Figure 2). Third, the hypothesized relations between the components of the framework were at least partially confirmed (see also Figure 1). Finally, the present study provided at least partial evidence for possible future additions to the framework.

In line with literature, multicultural personality traits were positively related to both the ethnocentric–ethnorelative worldview continuum [39,40], as well as multidimensional cultural intelligence [41,42]. Closer inspection of the framework components revealed that individuals with an open mind have a more ethnorelative disposition, while also showing better cultural capabilities through a higher score on motivation, cognition, and metacognition. Our findings also indicated that culturally empathic individuals showed a higher cultural intelligence through higher scores on motivation, metacognition, and behavior. As such, these two multicultural personality traits, open mindedness, and cultural empathy, seem to cover the full spectrum of cultural intelligence dimensions. These results were also largely in line with literature as the motivational dimension was pointed out as the key dimension that was positively related to the personality traits [41,42].

Moreover, also in line with the literature, individuals with a more ethnorelative disposition showed better intercultural capabilities [50,51]. Indeed, analogous to the cultural empathy trait, an individual’s ethnorelative disposition was related positively to the motivation, metacognition, and behavior dimension of the individual’s cultural intelligence.

Higher levels of cultural intelligence also predicted higher levels of cultural self-efficacy. These findings were also largely in line with literature [52,58,64,68]. An individual with higher scores on the motivation and cognition dimension of cultural intelligence showed higher levels of cultural self-efficacy through higher scores on the process, mix, cope, and understanding dimensions and on the process, cope, understanding, and language dimensions respectively.

Finally, the present study also explored possible extensions of the framework. First, four multicultural personality traits had a direct effect on intercultural effectiveness. (a) Open minded, (b) emotionally stable, (c) culturally empathic individuals or individuals that take (d) social initiative also showed higher levels of self-efficacy across all (sub)dimensions that was not mediated fully by the other components of the framework. Although the open mindedness trait thus represented an influential node in the framework with seven outgoing pathways, it was not the only dimension of multicultural personality that had a direct effect on cultural self-efficacy. Although we did not find a direct effect of flexibility which is at odds with literature [42], our results are largely in line with literature as the present study shows direct effects regarding four out of five multicultural personality traits on intercultural effectiveness [21,74,75]. Despite literature already features reports of direct effects regarding all five traits, these reports are inconclusive in pinpointing a trait of which the positive effect on intercultural effectiveness is consistent over situations. For the time being, we therefore advise to refrain from extending the existing framework with direct pathways from intercultural traits to intercultural effectiveness until more research on the matter is presented.

Second, the present study also reported some partial evidence that an ethnorelative world view is directly related to intercultural effectiveness through a larger understanding. This finding seems plausible that a broader view on the world evokes greater understanding of other cultures. The finding is also further supported by reports in literature of an ethnocentric view that evokes less understanding [76,77]. However, we again evaluate the existing evidence from literature and from the present study as insufficient to warrant an extension of the existing framework until more research is presented. Still, we do advocate testing the presence of direct pathways between (a) intercultural traits and effectiveness and (b) intercultural world views and effectiveness in future studies, as specific samples can always cause additional effects, above and beyond the current integrative framework.

Third and finally, analyses also revealed possible recursive pathways of self-efficacy to intercultural capabilities. Although these pathways can prove a worthwhile addition to the framework in the future, more theoretical and practical research needs to be conducted before such framework adaptations are warranted. At the moment, we consider the evidence too limited and too divergent (i.e., four possible pathways between cultural intelligence subdimensions and four possible pathways from self-efficacy subdimensions to cultural intelligence subdimensions) to suggest a specific recurrent pathway setup.

Summarizing our findings regarding the framework pathways, the main pathway in the framework from traits to effectiveness leads from open mindedness and cultural empathy (over an ethnorelative disposition) to cultural intelligence and ultimately to cultural self-efficacy, affecting all five self-efficacy subdimensions. In this indirect pathway, the motivation dimension of cultural intelligence is the first key node, as the node receives three pathways and is also sending out four pathways toward cultural self-efficacy. The second key node is the cognitive dimension of cultural intelligence, which receives only one pathway, but is also sending out four pathways toward cultural self-efficacy. This evidence confirms that knowledge and motivation form the prime gateway toward effective intercultural behavior [41,42,58,74,75].

### 4.1. Implications for Practice

The empirical validation of the framework can have important consequences for interventions that want to increase the effectivity of individuals in intercultural health situations by targeting specific components of intercultural competence. To obtain a basic understanding of an individual’s potential for intercultural effective behavior, a measurement of multicultural personality can serve as a baseline at the start of an intervention or education in health care. The specific results of the present study seem to suggest that researchers and practitioners should focus on the traits of open mindedness and cultural empathy. However, as traits are less suited for actual interventions that want to change behavior due to their higher stability [37,38], we instead advise to focus on the key node of motivation and cognition to a lesser extent for actual intervention. Indeed, both the present study and literature already indicate that the relation between cultural intelligence (motivation and cognition) and effective intercultural behavior is fairly strong as already stated. Moreover, cultural intelligence is quite malleable, making for an excellent candidate to target directly in future training, interventions, and identifying best practices, ultimately improving the intercultural effectiveness of individuals [19,58].

As a practical example of a future intervention, health care education can start by making students aware that motivated and educated practitioners will treat patients with a different cultural background more fairly and more effectively. This awareness can be complemented with a training that focuses on familiarizing students with important cultural phenomena and learning them how to incorporate these phenomena in treatment procedures. For instance, patients that adhere to the ways of Islam are not allowed to eat from sunrise to sunset during Ramadan. As a consequence, such a matter of faith can influence the diet of the patient during recovery. Health practitioners have to ensure that such nutritional issues are (at the very least) the subject of dialogue between practitioners and patients. In such a way, openminded responses to patient questions, supported by practitioner motivation and knowledge, can go a long way in making therapy and therapy outcomes during recovery more effective across patients with different cultural backgrounds, thus ensuring more equity in health care.

### 4.2. Limitations and Future Research

First, the theoretical framework clearly fits our data quite well [18]. Despite the good fit between the current empirical data and the proposed theoretical framework, we do acknowledge that we cannot rule out the possibility of additional components. For instance, patient/practitioner diversity is not limited to cultural aspects like ethnicity, but can also include gender or generation variables like the Six G’s Approach [91]. However, such an investigation into additional components is beyond the scope of the present study as the present study primarily focused on exploring the component relations within the framework. Second, the framework should be replicated using different conceptualizations, measures, and population samples of future health care practitioners. Although we have used specific conceptualizations of the core constructs, the present study does replicate the internal structure of four independent conceptualizations and instruments, while also providing empirical verification for the hypothesized framework relations. As such, we are confident that our empirical verification can serve as a baseline for future research. Third, our study makes use of a proxy of real world behavior. By using multidimensional cultural self-efficacy, we were able to also pinpoint how the different components of intercultural competence were related to intercultural self-efficacy. Future research should however include real-time behavior work adjustment, early withdrawal from intercultural jobs, the number of intercultural friends, inclusion policies on staff recruiting, an interest in politics and (a lack of) contact, and cooperation in both immigrant and non-immigrant students [25,47,48]. Fourth, the sample consisted of a sample of mostly future practitioners. Although such a sample validates our results toward education and previews the nature of health care practitioners in the near future, toward actual practice we do advocate some caution, as the students are not yet (fully) active. Finally, the framework should also be tested comparing different settings both inside and outside of health care. Although our hypotheses on the main components were confirmed, a health care setting could have different important (sub-) dimensions compared to housing or work settings. Barring the evidence on the motivational dimension from both literature and the present study, we therefore acknowledge that some (sub) dimensional pathways could shift depending on the setting of future studies. Of course, subsequent practical interventions should also take into account such shifted pathways. We therefore advise to always verify the framework as a baseline for practical interventions. To conclude, given the results of the present study regarding the construct validity and hypothesized relations of the components, we are cautiously optimistic that the empirically verified integrative framework is a robust starting point for future studies on intercultural competence and effectiveness.

## 5. Conclusions

In conclusion, the present study empirically verifies an integrative framework of intercultural competence and effectiveness. Intercultural capabilities remain the major gateway toward more effective intercultural behavior, with motivation and cognition acting as the key nodes in the framework. These dimensions are thus an excellent target for training, practical interventions, and identifying best practices, ultimately supporting greater intercultural effectiveness and more equity in health care.

## Figures and Tables

**Table 1:** Correlation matrix.

	*M*	*SD*	EC-ER	MPQ-CE	MPQ-FX	MPQ-SI	MPQ-ES	MPQ-OP	CSE-process	CSE-mix	CSE-cope	CSE-US	CSE-LAN	CSE	CQ-MOT	CQ-COG	CQ-META	CQ-BEH	CQ
EC-ER	39.99	8.2	1	0.181 **	0.153 **	0.066	0.066	0.398 **	0.308 **	0.296 **	0.021	0.362 **	0.075 *	0.319 **	0.470 **	0.121 **	0.256 **	0.194 **	0.371 **
MPQ-CE	33.42	3.44		1	−0.070 *	0.261 **	−0.062	0.387 **	0.338 **	0.265 **	0	0.303 **	0.127 **	0.302 **	0.415 **	0.144 **	0.229 **	0.238 **	0.366 **
MPQ-FX	19.62	5.66			1	0.099 **	0.257 **	0.148 **	0.064	0.141 **	0.003	0.108 **	0.043	0.113 **	0.101 **	0.05	−0.025	−0.047	0.033
MPQ-SI	26.58	5.13				1	0.273 **	0.275 **	0.224 **	0.288 **	0.037	0.130 **	0.134 **	0.249 **	0.145 **	0.112 **	0.081*	0.022	0.136 **
MPQ-ES	24.66	5.71					1	0.218 **	0.108 **	0.173 **	0.141 **	0.062	0.101 **	0.180 **	0.073 *	0.116 **	−0.052	−0.146 **	0.01
MPQ-OP	27.63	4.22						1	0.503 **	0.434 **	0.145 **	0.365 **	0.298 **	0.512 **	0.518 **	0.303 **	0.291 **	0.167 **	0.471 **
CSE-process	18.06	2.67							1	0.521 **	0.152 **	0.498 **	0.358 **	0.721 **	0.538 **	0.386 **	0.238 **	0.187 **	0.502 **
CSE-mix	3.52	4.45								1	0.306 **	0.446 **	0.349 **	0.829 **	0.559 **	0.230 **	0.191 **	0.158 **	0.414 **
CSE-cope	11.61	3.42									1	0.111 **	0.147 **	0.534 **	0.132 **	0.147 **	−0.033	−0.045	0.083 *
CSE-US	18.31	3.09										1	0.296 **	0.682 **	0.524 **	0.390 **	0.233 **	0.204 **	0.502 **
CSE-LAN	9.51	2.56											1	0.587 **	0.240 **	0.315 **	0.156 **	0.076 *	0.302 **
CSE	88.02	11.05												1	0.598 **	0.413 **	0.226 **	0.169 **	0.524 **
CQ-MOT	23.31	3.17													1	0.279 **	0.311 **	0.270 **	0.668 **
CQ-COG	17.17	3.99														1	0.186 **	0.155 **	0.651 **
CQ-META	21.16	3.34															1	0.557 **	0.731 **
CQ-BEH	21.4	2.9																1	0.682 **
CQ	83.05	9.13																	1

Note: MPQ = multicultural personality questionnaire, CE = cultural empathy, FX = flexibility, SI = social initiative, ES = emotional stability, OP = open mindedness, EC-ER = ethnocentric–ethnorelative continuum, CQ = cultural intelligence, MOT = motivation, COG = cognition, META = meta-cognition, BEH = behavior, CSE = cultural self-efficacy, US = understanding, LAN = language. * *p* < 0.05, ** *p* < 0.01.

**Table 2:** Regressions from model 1.

H	Regression	*E*	*SE*	*z*	*p*	ML
	EC-ER	~				
H1	MPQ-CE	0.18	0.11	1.55	0.121	0.06
H1	MPQ-FX	0.22	0.06	3.43	0.001	0.11
H1	MPQ-SI	−0.12	0.07	−1.67	0.096	−0.06
H1	MPQ-ES	−0.05	0.07	−0.77	0.441	−0.03
H1	MPQ-OP	0.99	0.09	10.66	0.000	0.38
	CQ	~				
H2	MPQ-CE	0.17	0.03	6.03	0.000	0.20
H2	MPQ-FX	−0.01	0.02	−0.62	0.537	−0.02
H2	MPQ-SI	0.00	0.02	−0.06	0.952	0.00
H2	MPQ-ES	−0.03	0.02	−1.81	0.070	−0.06
H2	MPQ-OP	0.24	0.03	9.33	0.000	0.33
	CQ	~				
H3	EC-ER	0.06	0.01	6.69	0.000	0.21
	CSE-TOT	~				
H4	CQ	0.40	0.04	10.79	0.000	0.35
H5	MPQ-CE	0.05	0.03	1.56	0.120	0.05
H5	MPQ-FX	0.02	0.02	0.92	0.358	0.03
H5	MPQ-SI	0.06	0.02	2.95	0.003	0.09
H5	MPQ-ES	0.06	0.02	2.99	0.003	0.09
H5	MPQ-OP	0.22	0.03	7.48	0.000	0.26
H6	EC-ER	0.02	0.01	2.07	0.038	0.06

Note: H = hypothesis, H1 = higher scores on intercultural traits predict a more ethnorelative world view, H2 = higher scores on intercultural traits predict higher scores on intercultural capabilities, H3 = a more ethnorelative world view predicts higher scores on intercultural capabilities, H4 = higher scores on intercultural capabilities predict higher scores on intercultural effectiveness, H5 = higher scores on intercultural traits directly predict higher scores on intercultural effectiveness, H6 = a more ethnorelative world view directly predicts higher scores on intercultural effectiveness. MPQ = multicultural personality questionnaire, CE = cultural empathy, FX = flexibility, SI = social initiative, ES = emotional stability, OP = open mindedness, EC-ER = ethnocentric–ethnorelative continuum, CQ = cultural intelligence, MOT = motivation, COG = cognition, META = meta-cognition, BEH = behavior, CSE = cultural self-efficacy, TOT = Total score of all five subscales, *E* = estimate, *SE* = standard error of the estimate, *z* = normalized estimate, *p* = result of the statistical test on the *z*–score to reject the null-hypothesis of *z* = 0, ML = model loading. The dependent variables of the regressions are indicated in italic.

**Table 3:** Regressions from Model 2.

H	Regression	*E*	*SE*	*z*	*p*	ML
	EC-ER	~				
H1	MPQ-FX	0.19	0.06	3.01	0.003	0.10
H1	MPQ-OP	1.00	0.08	12.07	0.000	0.38
	CQ	~				
H2	MPQ-CE	0.19	0.03	6.69	0.000	0.21
H2	MPQ-OP	0.22	0.02	9.08	0.000	0.31
	CQ	~				
H3	EC-ER	0.06	0.01	6.70	0.000	0.21
	CSE	~				
H4	CQ	0.42	0.04	11.35	0.000	0.36
H5	MPQ-SI	0.07	0.02	3.38	0.001	0.10
H5	MPQ-ES	0.05	0.02	3.02	0.003	0.09
H5	MPQ-OP	0.23	0.03	8.26	0.000	0.27
H6	EC-ER	0.02	0.01	2.19	0.029	0.07

Note: H = hypothesis, H1 = high scores on intercultural traits predict an ethnorelative world view, H2 = higher scores on intercultural traits predict higher scores on intercultural capabilities, H3 = a more ethnorelative world view predicts high scores on intercultural capabilities, H4 = higher scores on intercultural capabilities predict higher scores on intercultural effectiveness, H5 = higher scores on intercultural traits directly predict higher scores on intercultural effectiveness, H6 = a more ethnorelative world view directly predicts higher scores on intercultural effectiveness. MPQ = multicultural personality questionnaire, CE = cultural empathy, FX = flexibility, SI = social initiative, ES = emotional stability, OP = open mindedness, EC-ER = ethnocentric–ethnorelative continuum, CQ = cultural intelligence, MOT = motivation, COG = cognition, META = meta-cognition, BEH = behavior, CSE = cultural self-efficacy, *E* = estimate, *SE* = standard error of the estimate, *z* = normalized estimate, *p* = result of the statistical test on the *z*–score to reject the null–hypothesis of *z* = 0, ML = model loading. The dependent variables of the regressions are indicated in italic.

**Table 4:** Regressions from Model 3b.

H	Regression	*E*	*SE*	*z*	*p*	ML
	EC-ER	~				
H1	MPQ-OP	1.00	0.08	12.07	0.000	0.38
H1	MPQ-FX	0.19	0.06	3.01	0.003	0.10
	CQ-MOT	~				
H2	MPQ-CE	0.30	0.04	8.40	0.000	0.24
H2	MPQ-OP	0.30	0.03	9.67	0.000	0.30
H3	EC-ER	0.12	0.01	1.51	0.000	0.31
	CQ-COG	~				
H2	MPQ-OP	0.38	0.04	9.24	0.000	0.30
	CQ-META	~				
H2	MPQ-CE	0.17	0.04	3.85	0.000	0.13
H2	MPQ-OP	0.17	0.03	5.02	0.000	0.16
H3	EC-ER	0.07	0.01	4.85	0.000	0.17
	CQ-BEH	~				
H2	MPQ-CE	0.23	0.04	6.23	0.000	0.21
H3	EC-ER	0.05	0.01	4.57	0.000	0.15
	SE-process	~				
H4	CQ-MOT	0.31	0.03	9.77	0.000	0.31
H4	CQ-COG	0.16	0.02	7.49	0.000	0.21
H5	MPQ-CE	0.11	0.04	3.17	0.002	0.09
H5	MPQ-OP	0.25	0.03	7.53	0.000	0.25
	SE-mix	~				
H4	CQ-MOT	0.47	0.03	14.51	0.000	0.45
H5	MPQ-OP	0.15	0.03	4.63	0.000	0.14
H5	MPQ-SI	0.14	0.02	6.39	0.000	0.17
	SE-cope	~				
H4	CQ-MOT	0.16	0.06	2.95	0.003	0.10
H4	CQ-COG	0.13	0.04	3.15	0.002	0.10
H5	MPQ-ES	0.11	0.04	2.74	0.006	0.09
	SE-US	~				
H4	CQ-MOT	0.38	0.04	9.85	0.000	0.33
H4	CQ-COG	0.24	0.03	9.26	0.000	0.26
H5	MPQ-CE	0.15	0.04	3.54	0.000	0.11
H6	EC-ER	0.07	0.01	5.14	0.000	0.16
	SE-LA	~				
H4	CQ-COG	0.30	0.04	7.15	0.000	0.23
H5	MPQ-OP	0.36	0.05	6.68	0.000	0.22

Note: H = hypothesis, H1 = high scores on intercultural traits predict an ethnorelative world view, H2 = higher scores on intercultural traits predict higher scores on intercultural capabilities, H3 = a more ethnorelative world view predicts high scores on intercultural capabilities, H4 = higher scores on intercultural capabilities predict higher scores on intercultural effectiveness, H5 = higher scores on intercultural traits directly predict higher scores on intercultural effectiveness, H6 = a more ethnorelative world view directly predicts higher scores on intercultural effectiveness. MPQ = multicultural personality questionnaire, CE = cultural empathy, FX = flexibility, SI = social initiative, ES = emotional stability, OP = open mindedness, EC-ER = ethnocentric–ethnorelative continuum, CQ = cultural intelligence, MOT = motivation, COG = cognition, META = meta-cognition, BEH = behavior, CSE = cultural self-efficacy, US = understanding, LAN = language, *E* = estimate, *SE* = standard error of the estimate, *z* = normalized estimate, *p* = result of the statistical test on the *z*–score to reject the null–hypothesis of *z* = 0, ML = model loading. The dependent variables of the regressions are indicated in italic.

**Table 5:** Explained variance in endogenous variables of Model 3b.

	CQ-MOT	CQ-COG	CQ-META	CQ-BEH	EC-ER	SE-Process	SE-Mix	SE-Cope	SE-US	SE-LAN
R^2^	0.397	0.092	0.116	0.076	0.167	0.384	0.361	0.035	0.339	0.135

Note: EC-ER = ethnocentric–ethnorelative continuum, CQ = cultural intelligence, MOT = motivation, COG = cognition, META = meta-cognition, BEH = behavior, CSE = cultural self-efficacy, US = understanding, LAN = language.

## Data Availability

The data that support the findings of this study are available on request from the corresponding author S.S. The data are not publicly available due to them containing information that could compromise research participant privacy/consent. However, the variance–covariance matrices of the data is provided online in .txt format, allowing for full verification and replication of the complete study.

## Supplementary Materials

The following supporting information can be downloaded at: https://www.mdpi.com/article/10.3390/ijerph19084490/s1, File S1: variance-covariance matrix full.txt for replication of the CFA analyses, File S2: variance-covariance matrix sub.txt for the replication of the structural SEM analyses.
